# Bisphenol S Impaired Human Granulosa Cell Steroidogenesis in Vitro

**DOI:** 10.3390/ijms21051821

**Published:** 2020-03-06

**Authors:** Sarah Amar, Aurélien Binet, Ophélie Téteau, Alice Desmarchais, Pascal Papillier, Marlène Z. Lacroix, Virginie Maillard, Fabrice Guérif, Sebastien Elis

**Affiliations:** 1 PRC, INRAE, CNRS, Université de Tours, IFCE, 37380 Nouzilly, France; ssa.amar@sfr.fr (S.A.); aurelien.binet@univ-tours.fr (A.B.); Ophelie.Teteau@inrae.fr (O.T.); alice.desmarchais@inrae.fr (A.D.); virginie.maillard@inrae.fr (V.M.); guerif@med.univ-tours.fr (F.G.); 2 Service de Chirurgie pédiatrique viscérale, urologique, plastique et brûlés, CHRU de Tours, 37000 Tours, France; 3 Therapeutic Innovations and Resistance (INTHERES), Université de Toulouse, INRAE, ENVT, 31076 Toulouse, France; 4 Service de Médecine et Biologie de la Reproduction, CHRU de Tours, 37000 Tours, France

**Keywords:** endocrine disruptors, Bisphenol S, women, granulosa cells, steroidogenesis, proliferation

## Abstract

Bisphenol S (BPS) is a structural analog of the endocrine disruptor bisphenol A (BPA); it is the main BPA replacement in the plastics industry. Previous studies have shown that BPA and BPS exhibit similar effects on reproduction in fish and rodent species. BPS reportedly alters steroidogenesis in bovine granulosa cells. Luteinised granulosa cells collected from 59 women who were undergoing an in vitro fertilization procedure were cultured for 48 h in the presence or absence of BPS (10 nM, 100 nM, 1 µM, 10 µM or 50 µM). BPS exposure was investigated by assessing follicular fluids from these 59 women for their BPS content. Culture medium, cells, total messenger RNA (mRNA) and total protein extracted from the luteinised granulosa cells were examined for oestradiol and progesterone secretion, cellular proliferation, viability, gene expression, steroidogenic enzyme expression and cell signaling. BPS was measured in follicular fluids using mass spectrometry. Exposure of granulosa cells to 10 or 50 µM BPS for 48 h induced a 16% (*p* = 0.0059) and 64% (*p* < 0.0001) decrease, respectively, in progesterone secretion; 50 µM BPS decreased oestradiol secretion by 46% (*p* < 0.0001). Ten µM BPS also tended to reduce CYP11A1 protein expression by 37% (*p* = 0.0947) without affecting HSD3B1 and CYP19A1 expression. Fifty µM BPS increased ERRγ expression. Environmental levels of BPS (nanomolar range) did not induce changes in steroidogenesis in human granulosa cells. The effects of BPS were observed after only 48 h of BPS exposure. These acute effects might be similar to chronic effects of physiological BPS levels.

## 1. Introduction

An increase in human infertility in western countries has led researchers to question the impact of environmental factors, especially endocrine disruptors. Previous studies reported positive correlations between the levels of pollutants in female follicular fluids with the outcome of in vitro fertilisation (IVF) and embryo transfer [1,2]. Bisphenol A (BPA) is an endocrine disruptor and a widespread plasticiser that is mainly used in polycarbonate monomers, epoxy resins and thermal papers due to its heat resistance and elasticity properties. It was extensively used for more than 50 years in the industry, particularly to produce food containers, baby bottles and metal cans. However, it was also used in medical devices, soaps, lotions, shampoo, nail polish, sunscreen and even toys [3,4]. Humans are exposed to BPA mainly through diet, due to container-content transfer [5,6,7], but also through indoor dust inhalation [8] and the transcutaneous route [9].

Widespread BPA use led to its detection in 95% of patient urine samples in the United States at concentrations ≥ 0.1 ng/mL (0.44 nM; [10]), with an average urine and blood concentration of 1 to 3 ng/mL (4–13 nM; [4]). BPA has also been detected in amniotic fluid (1–9 ng/mL), follicular fluid (2.4 ± 0.8 ng/mL), neonatal, placental and foetal blood (2.2 ± 1.8 ng/mL), human breast milk (approximately 1 ng/mL) and adipose tissue from 1.80 to 12 ng/g [11,12,13,14]. These data highlight exposure windows that occur during pregnancy or infancy.

Previous studies investigated the deleterious effects of BPA on health [12,15,16] and evidenced that low concentrations (in the nanomolar range) are associated with obesity, cardiovascular diseases [16,17], type 2 diabetes [17,18] and alterations in reproductive function [19]. BPA exhibits a weak oestrogenic activity [20] due to a low affinity for the oestrogen receptor (ER) α and ERβ [21,22]. Moreover, the highest urinary BPA concentrations in women undergoing IVF are associated with decreased oocyte number and quality and reduced oestradiol levels [23,24,25]. BPA also reportedly disrupts steroid production in rat and porcine granulosa cells [26,27,28,29]. BPA decreases progesterone secretion in both rat and human granulosa cells [30,31,32].

Several studies demonstrated the gradual increase in the human unregulated BPA analog exposure, namely Bisphenol S (BPS), because of BPA prohibition [33,34] in some countries [35]. BPS is therefore now detected in urine at the same concentration range as BPA (0.02–21 ng/mL or 0.09–91 nM; [36]). Due to their structural analogy, it is suggested that BPS and BPA exhibit similar properties and adverse health effects. Some studies on fish and rodent species reported BPS effects on germinal cells and endocrine function that are similar to BPA (reviewed in [37]). Indeed, BPS reduces egg production and sperm count [38] and egg hatchability in zebrafish [39]. BPS also affects oocyte quality in mice [40] and testis oxidative stress and spermatogenesis in rats [41]. Moreover, BPS alters steroidogenesis in bovine granulosa cells [42]. Since rodent species previously showed resistance to bisphenols [4], it is necessary to assess BPS effects in human cells.

We hypothesised that BPS would exhibit deleterious effects in human granulosa cells. The aim of the present study was therefore to investigate the acute in vitro effects of BPS on the steroidogenic functions of primary human granulosa cells (HGC) and elucidate its mechanisms of action. We also assessed HGC environmental exposure to BPS by measuring the metabolised form of BPS, namely BPS glucuronide (BPSG), in female follicular fluid.

## 2. Results

### 2.1. Cell Viability

**Figure 1:**
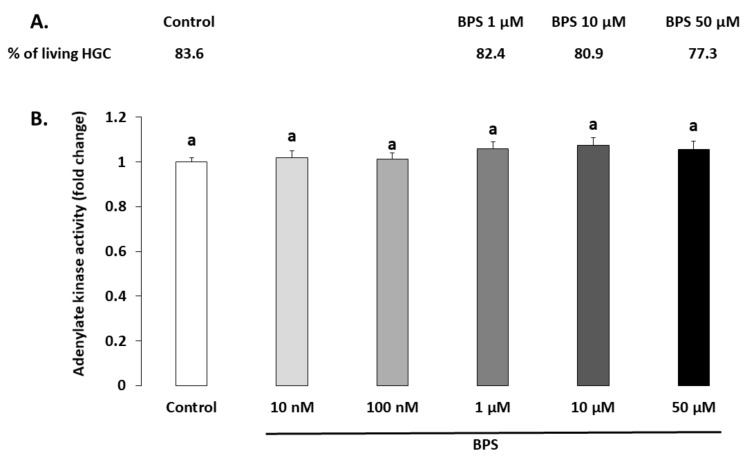
Effect of Bisphenol S (BPS) on human granulosa cell (HGC) viability. HGC underwent 48-h culture in the presence or absence of BPS (10 nM, 100 nM, 1 µM, 10 µM or 50 µM). (**A.**) HGC viability was assessed using the Live/Dead Viability/Cytotoxicity Kit for mammalian cells. The results of three independent experiments per condition are expressed as the percentage of living cells in each condition. (**B.**) HGC viability was assessed on spent 48-h culture media using the Bioluminescence Cytotoxicity Assay Kit. Data are expressed as the mean relative light units ± standard error of the mean (SEM) of six independent cultures. Bars with different superscripts indicate a significant difference (*p* ≤ 0.05).

The BPS effects on cell viability was investigated through both HGC live/dead staining (Figure 1A) and adenylate kinase assay in spent media (Figure 1B) after 48-h treatment. The living HGC rate evaluated using live/dead staining was 83.6% for the control condition and was not affected by 1, 10 or 50 µM BPS (82.4%, *p* = 0.191; 80.9%, *p* = 0.91; and 77.3%, *p* = 0.323, respectively). HGC viability was also assessed by measuring adenylate kinase activity in spent HGC culture media (Figure 1B). There was no significant difference in adenylate kinase activity between the control and any BPS condition (*p* = 0.182).

### 2.2. Cell Proliferation

**Figure 2:**
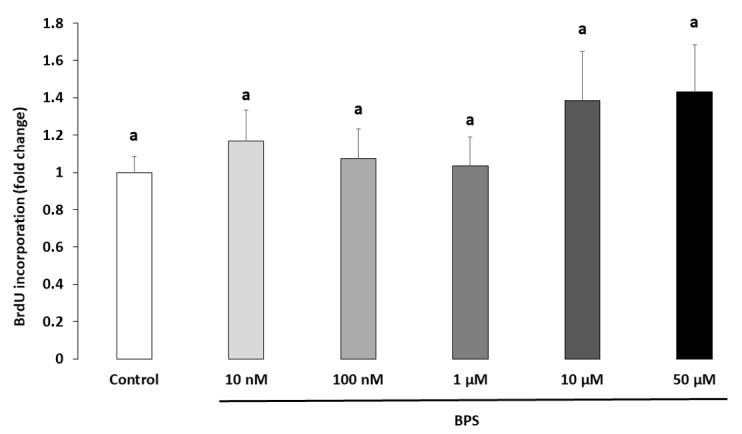
Effect of Bisphenol S (BPS) on human granulosa cell (HGC) cellular proliferation. HGC underwent 48-h culture with 10 µM bromodeoxyuridine/5-bromo-2’-deoxyuridine (BrdU), in the presence or absence of BPS (10 nM, 100 nM, 1 µM, 10 µM or 50 µM). The cell proliferation was normalised to the control condition of each culture. The results are expressed as the mean ± standard error of the mean (SEM) of five independent experiments with four replicates per condition. Bars with different superscripts indicate a significant difference (*p* ≤ 0.05).

Cell proliferation was measured using BrdU incorporation after 48 h HGC culture. There were no differences in cell proliferation at any BPS concentration compared to the control (Figure 2).

### 2.3. Steroidogenesis

**Figure 3:**
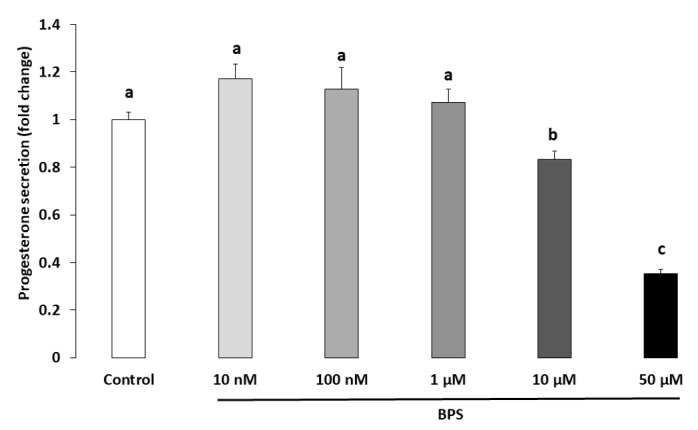
Effect of Bisphenol S (BPS) on human granulosa cell (HGC) progesterone secretion. HGC underwent 48-h culture in the presence or absence of BPS (10 nM, 100 nM, 1 µM, 10 µM or 50 µM). The progesterone concentration was measured in culture media, and its value was normalised by the protein concentration in each well. Data are expressed as ng progesterone per µg protein and normalised to the control condition. The results of seven independent experiments are presented as the mean ± standard error of the mean (SEM). Bars with different superscripts indicate a significant difference (*p* ≤ 0.05).

**Figure 4:**
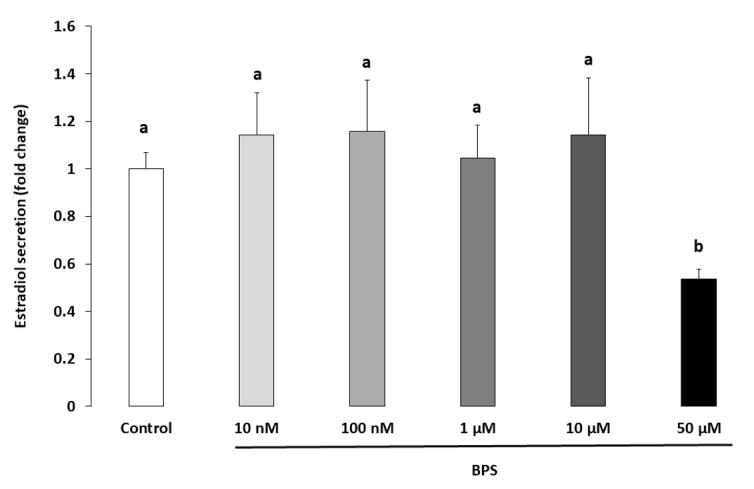
Effect of Bisphenol S (BPS) on human granulosa cell (HGC) oestradiol secretion. HGC underwent 48-h culture in the presence or absence of BPS (10 nM, 100 nM, 1 µM, 10 µM or 50 µM). The oestradiol concentration was measured in culture media, and its value was normalised by the protein concentration in each well. Data are expressed as pg oestradiol per µg protein and normalised to the control condition. The results of seven independent experiments are presented as mean ± standard error of the mean (SEM). Bars with different superscripts indicate a significant difference (*p* ≤ 0.05).

Progesterone and oestradiol were measured in spent culture media after a 48-h BPS treatment (Figure 3 and Figure 4, respectively). Ten µM of BPS decreased progesterone by 16% (*p* = 0.0059) compared with the control condition. At 50 µM BPS, progesterone was decreased by 64% compared to control (*p* < 0.0001; Figure 3). Fifty µM BPS reduced oestradiol secretion by 46% compared to control (*p* < 0.0001; Figure 4). There were no differences in progesterone or oestradiol secretion at any other BPS concentration.

### 2.4. Protein Expression

**Figure 5:**
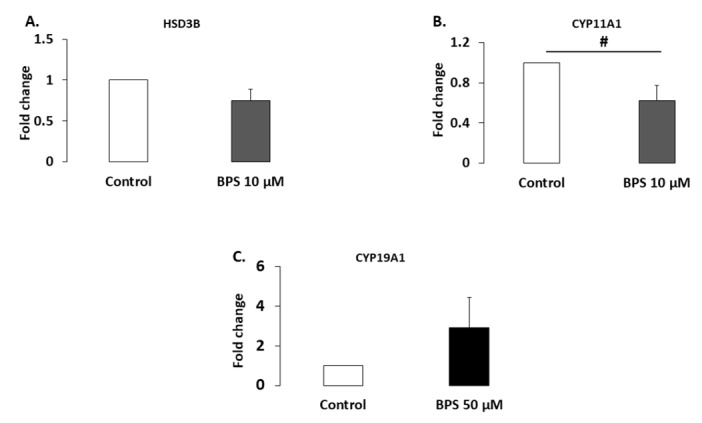
Effect of Bisphenol S (BPS) on steroidogenic enzyme expression. HGC underwent 48-h culture in the presence or absence of BPS (10 nM, 100 nM, 1 µM, 10 µM or 50 µM). Proteins were then extracted and separated using electrophoresis on 4–12% (*w/v*) sodium dodecyl sulphate polyacrylamide gels. After electrotransfer to nitrocellulose membranes, the proteins were probed with HSD3B (**A**), CYP11A1 (**B**), CYP19A1 (**C**) and VCL antibodies. Bands on the blots were quantified, and the data are expressed in arbitrary units as the ratio of HSD3B (*n* = 5), CYP11A1 (*n* = 3) or CYP19A1 (*n* = 5) to VCL. The results are expressed relative to the control as the mean ± standard error of the mean (SEM). # indicates a tendency (*p* < 0.10).

HSD3B1 and CYP11A1 are steroidogenic enzymes involved in progesterone synthesis. Their protein expression levels were analysed using western blotting in HGC treated with 10 µM BPS, a concentration that decreased progesterone secretion (Figure 3). Ten µM of BPS treatment showed a tendency to decrease CYP11A1 expression levels (37.7%; *p* = 0.0947; Figure 5B), but it did not affect HSD3B1 expression (*p* = 0.124; Figure 5A). CYP19A1 is a steroidogenic enzyme involved in oestradiol secretion. Its expression level was therefore investigated in HGC treated with 50 µM BPS, the concentration that reduced oestradiol secretion. This treatment did not affect CYP19A1 expression (*p* = 0.564; Figure 5C).

### 2.5. Gene Expression

**Figure 6:**
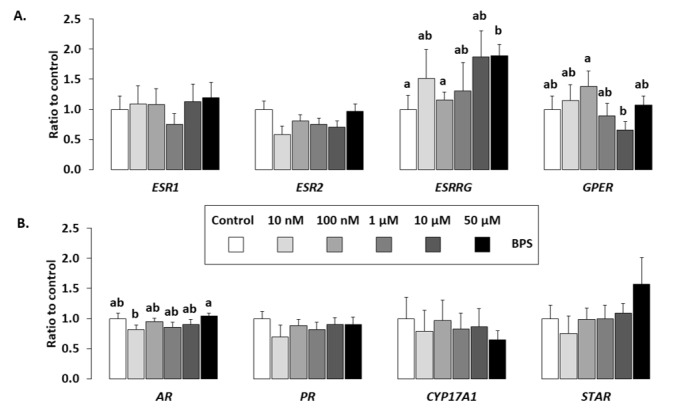
Effect of BPS on HGC gene expression. The expression of six hormonal receptors (oestrogen receptors (A): oestrogen receptor 1 [ESR1], oestrogen receptor 2 [ESR2], Oestrogen-related receptor γ [ESRRG] and G protein-coupled oestrogen receptor [GPER] and (B) androgen receptor [AR], progesterone receptor [PR]), and two genes involved in steroidogenesis (B: CYP17A1 and StAR), was determined in HGC after 48-h culture in complemented serum-free McCoy’s 5A media in the presence or absence of BPS (10 nM, 100 nM, 1 µM, 10 µM or 50 µM). Total messenger RNA (mRNA) was extracted from HGC and reverse transcribed, and real-time polymerase chain reaction (qPCR) was performed. The geometric mean of two housekeeping genes (Glyceraldehyde-3-phosphate dehydrogenase [GAPDH] and ribosomal protein L19 [RPL19]) was used to normalise gene expression. The results are expressed as mean ± standard error of the mean (SEM) of six independent cultures and normalised to the mean of control condition. No letter in common indicates a significant difference (*p* < 0.05).

Expression of eight genes (Table 1), including steroid and hormonal receptors (*ESR1, ESR2, ESRRG, GPER, AR* and *PR*) and genes involved in steroidogenesis (*CYP17A1* and *STAR*), was measured in HGC using qPCR after 48-h BPS treatment (Figure 6). *ESRRG* expression significantly 1.9-fold increased after BPS 50 µM treatment compared to the control (*p* < 0.0001). BPS treatment had no effect on the other analysed genes, when compared to the control.

### 2.6. Signalling Pathways

**Figure 7:**
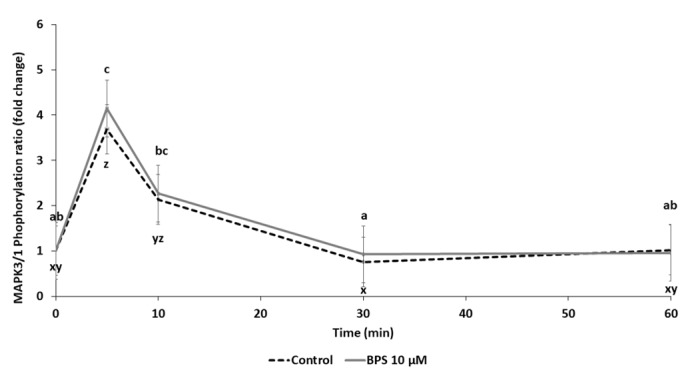
Western blot analysis of MAPK3/1 signalling pathway activation in human granulosa cell (HGC) after Bisphenol S (BPS) exposure. HGC underwent a time response (0 to 60 min culture) in the presence or absence of BPS 10 µM. Proteins were then extracted and separated using electrophoresis in 4–12% (*w/v*) sodium dodecyl sulphate polyacrylamide gels. After electrotransfer to nitrocellulose membranes, the proteins were probed with phospho-MAPK3/1 and total MAPK3/1 antibodies. Bands on the blots were quantified, and the data are expressed in arbitrary units as the ratio of phospho- to total MAPK3/1 as the mean ± standard error of the mean (SEM) of six independent experiments. Different letters indicate significant differences (*p* ≤ 0.05). abc letters correspond to differences in MAPK3/1 phosphorylation between 10 µM BPS timepoints, and xyz letters correspond to differences in MAPK3/1 phosphorylation between control timepoints.

MAPK3/1 signalling pathway was investigated after 5, 10, 30 and 60 min treatment in the presence or absence of 10µM BPS (Figure 7). In control cells (HGC treated with medium only), a transient 3.7-fold increase in MAPK3/1 phosphorylation was observed after 5 min (*p* < 0.0001). There was a similar 4.1-fold increase in MAPK3/1 phosphorylation after 5 min with 10 µM BPS. There was no difference in MAPK3/1 phosphorylation between 10 µM BPS and control treatments.

### 2.7. Female Follicular Fluid BPS Glucuronide Assays

**Figure 8:**
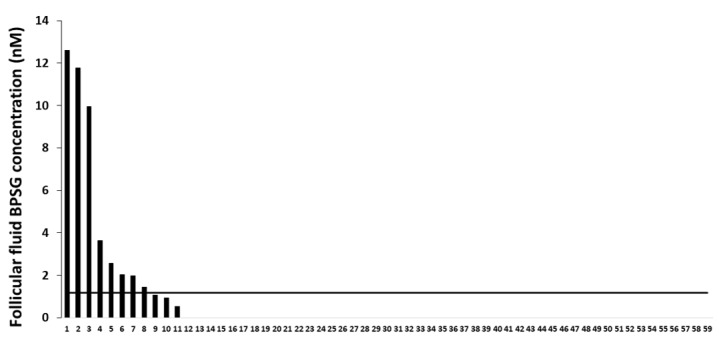
Female follicular fluid exposure to Bisphenol S (BPS). Follicular fluids from 59 women (in abscissa) were collected. Assays that measured BPS glucuronide (BPSG) were performed using ultraperformance liquid chromatography-tandem mass spectrometry (UPLC MS/MS). The limit of quantification (LOQ) value, presented with a horizontal black line, was 1.17 nM (0.5 ng/mL) for BPSG in female follicular fluid. The BPSG level is represented for each of the 59 women in nM.

Follicular fluid samples were collected from 59 women who underwent IVF procedure (Figure 8). BPS glucuronide was detected in 11 samples (18.6%). The concentration ranged from 0.5 to 12.6 nM, with an average of 4.4 ± 1.4 nM (1.9 ± 0.6 ng/mL).

## 3. Discussion

This study aimed to evaluate the effects of BPS, a structural analog of BPA used as its replacement, on HGC functions, especially HGC steroidogenesis. For the first time, we reported that BPS inhibited both progesterone and oestradiol secretion. BPS could affect progesterone secretion partially through a decrease in CYP11A1 secretion. BPS increased the expression of ERRγ. BPS was detected in 18.6% of the investigated female follicular fluid samples.

### 3.1. BPS Reduced HGC Progesterone and Oestradiol Secretion

In the present study, BPS significantly reduced HGC progesterone secretion. This effect is consistent with previous data from animal models. Indeed, BPS exposure (5 mg/kg body weight) decreases plasma progesterone in rats [43] and progesterone secretion in ewe GC and cumulus cells [44,45]. However, discrepancies among species or tested doses can occur. GC exposed to 10 µM BPS did not exhibit reduced progesterone secretion in swine [46] or cows [42]. This result may indicate a difference in sensitivity among species: women and ewes may be more sensitive to BPS effects compared to swine and cows. Comparatively, BPA also reduced progesterone secretion in female GC [32], rat GC [30,31] and swine GC [26,27,29]. Nevertheless, contrasting effects were reported between low (1 µM, increased progesterone secretion) and high (100 µM, decreased progesterone secretion) BPA concentrations in swine [26] and rat GC [28]. Progesterone plays a critical function in HGC. It is especially important because HGC were collected from pre-ovulatory follicles. HGC are therefore close to pre-luteal cells, and a decrease in progesterone secretion may affect the capacity to sustain embryo development, implantation and gestation.

The highest BPS concentration significantly reduced oestradiol secretion. This result is inconsistent with previous data in animal models, where BPS exposure increases oestradiol secretion in rat GC [43], ewe GC [44] and cow GC [42]. Nevertheless, as mentioned above, the fact that HGC were only collected from pre-ovulatory follicle (contrary to other animal models) could explain the observed discrepancies. Indeed, a similar decrease in oestradiol secretion was previously reported in HGC after BPA exposure [32], as well as in swine GC [27]. Such a decrease in oestradiol was also inversely correlated with plasma BPA levels in women [47]. In the present study, decreases in both progesterone and oestradiol occurred with high BPS concentrations, much higher compared to the level measured in female follicular fluids. However, the cells were exposed for only 48 h. The observed effects may also occur when exposing cells at a lower concentration but on a chronic basis.

We observed a 16% and 64% decrease in progesterone after 10 and 50 µM BPS treatment, respectively. We then compared the BPS effects reported in the present study with BPA effects reported in the literature in terms of their intensity. A study in HGC reported a 20% and 75% decrease in progesterone secretion after 2 and 20 µg/mL (8.8 and 88 µM) BPA treatment, respectively [32]. We found a 46% decrease in oestradiol secretion after 50 µM BPS treatment, while Mansur et. al reported a 60% decrease after 88 µM BPA treatment [32]. In both studies, treatment lasted for 48 h. These data suggest that BPS and BPA have a similar effect on human steroidogenesis in terms of intensity.

Moreover, as mentioned above, the BPS effects appeared to differ depending on the species. Indeed, HGC appear to be more sensitive to BPS treatment compared to other animal models, including swine [45] and cow GC [42]. On the contrary, HGC are similar in terms of sensitivity to progesterone secretion with ewe GC. In ovine granulosa cells, progesterone declined by 22% and 32% after 10 µM and 50 µM BPS treatment, respectively [44], data that are similar to the results obtained in the present paper. These findings confirm that the ewe is a relevant model to study human reproductive function in terms of effects and sensitivity of cells [48,49,50].

### 3.2. BPS Mechanisms of Action

The main function affected by BPS treatment was HGC steroidogenesis, namely progesterone and oestradiol secretion. BPS treatment reduced protein expression of CYP11A1, a steroidogenic enzyme involved in the transformation of cholesterol into pregnenolone. Pregnenolone is the precursor of progesterone, and thus a lower pregnenolone concentration may lead to reduced progesterone production and secretion, a hypothesis that is consistent with the data reported here. BPS did not affect HSD3B1, which is involved in the conversion of pregnenolone into progesterone. Other proteins should be investigated, for example, StAR, which is a cholesterol transporter. Moreover, there was no effect of BPS on CYP19A1, also called aromatase, a steroidogenic enzyme involved in oestradiol synthesis from testosterone. A previous study reported a decrease in aromatase and StAR correlated with BPA exposure that explains reduced oestradiol secretion [51]. These data suggest that BPS and BPA may affect oestradiol secretion through separate pathways. It would be interesting to assess the expression of HSD17B3, an enzyme involved in the conversion of androstenedione (present in the culture media) into testosterone, as changes in this enzyme may explain the observed reduction in oestradiol. In addition to expression, assessing the activity of enzymes could also highlight whether BPS affects steroidogenesis through these enzymes.

We also examined the MAPK3/1 signalling pathway. BPA reportedly exerts its effects at least partially through an increase in MAPK3/1 phosphorylation [52,53]. There was a transient increase in MAPK3/1 phosphorylation in both control and BPS-treated HGC. MAPK3/1 signalling pathway is involved in granulosa cell steroidogenesis [54]. Difference between the BPA and BPS effect on the MAPK3/1 signalling pathway were evidenced in ovine granulosa cells [44]. Nevertheless, these data suggest that the BPS effect on steroidogenesis is likely independent of the MAPK3/1 pathway, as it was reported in ovine GC. Among the numerous signalling pathways that were reported to be involved in BPA effects, future studies could focus on those that include phosphoinositide 3-kinase (PI3K)/Akt, which is involved in BPA effects in granulosa cells [55].

Concerning expression of the candidate genes assessed, only *ESRRG* had an increased expression after BPS treatment. *ESRRG* is expressed at a high level in placenta and kidney compared to ovary [56]. ERRγ is also able to strongly bind BPA; nevertheless, ERRγ roles are still poorly understood [56]. Moreover, bisphenols, like BPA, exhibit weak oestrogenic activity due to a low binding affinity to ESR1 and ESR2. Moreover, because HGC are oestradiol-producing cells, it is possible that the BPS effects observed in this study are unrelated to ESR1 and ESR2. Further studies should focus on potential mechanisms of action through other receptors, such as ESRRG for example, and assess whether BPS could also strongly bind to ERRγ, as BPA.

In the present study, there was no effect of BPS treatment on HGC viability and proliferation that could have secondarily explained the effects observed on hormone secretion. Considering the utilised cell model, namely HGC collected from women who were undergoing IVF procedure, the cells are exposed to ovarian stimulation treatment and cannot therefore be considered regular granulosa cells. As mentioned before, HGC are also closer to pre-luteal cells rather than regular granulosa cells.

### 3.3. BPS in Follicular Fluid

In the present study, BPSG was detected in the follicular fluid of 11/59 women. To our knowledge, this study is the first to report a BPSG assay in human follicular fluids. BPSG is one of the metabolised forms of BPS. The glucuronidation of BPS mainly occurs in the liver, and BPSG is then eliminated through urine. The advantage of measuring BPSG in a sample is to determine human exposure without the risk of the sample being contaminated by the environment or the tube. Indeed, a sample can be contaminated by BPS but not BPSG. Previous data on ewe oocyte quality reported a detrimental effect of BPS at a concentration as low as 10 nM [45], which corresponds to the level of BPSG reported in female follicular fluids in the present study. Therefore, these data raise the issue of human oocyte sensitivity to BPS treatment, which may be similar to the sensitivity observed in the ovine model. In this regard, BPS does not appear to be a safe replacement to BPA. Moreover, by measuring BPSG only in follicular fluid, the percentage of women exposed to BPS could be underestimated, especially if the ovarian follicle reduces the BPSG levels that reach the follicular fluids. Future analysis will therefore combine assays performed in follicular fluid with assays performed in urine.

Several limitations occurred in this study, related to the use of human granulosa cells from women undergoing IVF procedure. Indeed, great variability can occur between women. Cells were collected after women received ovarian stimulation treatment. HGC were also collected during the preovulatory stage and are therefore close to pre-luteal cells. Nevertheless, HGC underwent a short culture period and they were cultured without serum to slow their differentiation. Furthermore, the McCoy medium used for culture contained phenol red, which has oestrogenic properties and might not be ideal to evaluate endocrine disrupting properties of compounds such as bisphenols. Nevertheless, because HGC are oestradiol-producing cells. As oestradiol has stronger oestrogenic properties, it renders negligible the oestrogenic effect of phenol red. The effects of BPS were therefore studied in an oestrogenic environment, corresponding to physiological conditions. Here, we described the acute effects (48-h culture) of BPS on HGC steroidogenesis. We showed here that BPS reduced progesterone secretion and oestradiol secretion. These data suggested that BPS might not be a safe alternative for BPA, which could have a high relevance for human health and reproduction.

## 4. Materials and Methods

### 4.1. Participants, Biological Material and Ethical Approval

HGC were collected during oocyte retrieval from women who were undergoing IVF. This study was approved by The Ethics Committee on Research involving Human Subjects of Bretonneau University Hospital (Tours, France) (Research Project DC-2014-2285, 15/04/2014), and each patient had previously signed a consent form authorising the use of granulosa cells, which are normally discarded. All patients were included except women who were undergoing IVF for ovarian issues.

### 4.2. Chemicals and Antibodies

BPS was purchased from Sigma-Aldrich (Saint Quentin Fallavier, France). All other chemicals were obtained from Sigma-Aldrich, unless otherwise stated in the text. The rabbit antibody to 3-beta-hydroxysteroid dehydrogenase (HSD3B1) was purchased from Abgent (San Diego, California, USA). The goat antibodies to cytochrome P450 family 11 subfamily A member 1 (CYP11A1) was obtained from Santa Cruz Biotechnology (EUROMEDEX, Souffelweyersheim, France). The mouse monoclonal antibody to human vinculin (VCL clone hVIN-1) and rabbit antibody to cytochrome P450 family 19 subfamily A member 1 (CYP19A1) were purchased from Sigma-Aldrich. Rabbit polyclonal antibodies to rat P44/42 mitogen-activated protein kinase (MAPK 3/1) and human phospho-p44/42 MAPK (MAPK 3/1; Thr202/Tyr204) were obtained from Cell Signaling Technology (Ozyme, Saint Quentin Yvelines, France). Horseradish-peroxidase-conjugated anti-rabbit, anti-goat and anti-mouse IgG were purchased from Perkin Elmer (Courtaboeuf, France).

### 4.3. Isolation and Culture of HGC

HGC were recovered from oocyte punctures performed in 59 women who were undergoing IVF procedure (and therefore received an ovarian stimulation treatment). After centrifugation of the follicular fluid (5 min at 400 *g*), they were stored at −20 °C until analysis. The pellet, which contained the HGC, was resuspended in ACK haemolysis buffer (154.95 mM ammonium chloride, 9.99 mM potassium bicarbonate, 0.10 mM ethylenediaminetetraacetic acid [EDTA]) for 3 min at room temperature. Cells were then centrifuged (5 min, 400 *g*), and after the removal of the supernatant that contained the lysed red blood cells, the cells were washed in modified McCoy’s 5A serum-free medium, supplemented with 3 mM L-glutamine, 0.1% bovine serum albumin, penicillin/streptomycin (120 × 10^3^ UI/L penicillin; 120 mg/L streptomycin), 20 mM HEPES (pH 7.6), 96 nM 11βOH-4-androstenedione, bovine apo-transferrin (5 mg/L), 0.12 µM selenium and 1.74 nM insulin. Cells were then applied to a Percoll density medium (50% Percoll, 50% medium), and HCG were purified using centrifugation (30 min, 700 *g*). After washes in medium and centrifugation (5 min, 400 *g*), cells were resuspended in modified McCoy’s 5A and counted (after trypan blue staining) on a haemocytometer. Cells were incubated (100,000 living cells per well in 150 μL modified McCoy’s 5A, unless otherwise stated in the text; 96-well plate, BioLite, Thermo Fisher Scientific, Illkirch, France) and grown overnight in a humidified atmosphere that contained 5% CO_2_ in air at 37 °C.

HGC were treated the next day by replacement of the culture medium, in the presence or absence of BPS, including both environmental levels (10 nM and 100 nM) and supraenvironmental levels (1 μM, 10 μM or 50 μM), and the cells were cultured in a humidified atmosphere that contained 5% CO_2_ in air at 37 °C for 48 h for steroidogenesis, viability, cell proliferation and gene and protein expression experiments.

### 4.4. Cell Viability

HGC were cultured on Permanox® Nunc™ Lab-Tek™ Chamber Slide™ (Thermofischer scientific) (250,000 cells per well) in 250 µL modified McCoy’s. After 48-h treatment in the presence or absence of BPS (1 μM, 10 μM or 50 μM), the supernatants were collected to assess adenylate kinase activity, and cells were stained to assess the rate of living cells.

HGC viability was assessed using the Live/Dead Viability/Cytotoxicity Kit for mammalian cells (Life Technologies, Cergy Pontoise, France), according to the supplier’s instructions. Four fields per condition were counted (200 cells minimum per condition). The results are expressed as the percentage of the number of living (green) cells for three independent experiments.

Supernatants were stored at −20 °C until the adenylate kinase activity assay using the Bioluminescence Cytotoxicity Assay kit (MBL International, CliniSciences, Nanterre, France) was performed (according to the supplier’s recommendations). The plate was read using a Tristar^2^ S LB 942 Multimode Reader Luminometer (Berthold Technologies, Yvelines, France) and using MikroWin software (version 5.14, Labsis Laborsysteme GmbH, Neunkirchen-Seelscheid, Germany). The results are expressed in relative light units normalised to the control of each culture for six experiments, with two replicates per condition, and correspond to the average of the values obtained between 15 and 20 min of the reaction (maximum signal). The adenylate kinase activity is positively correlated to the dead cell rate.

### 4.5. HGC Proliferation

After 48-h treatment with 10 µM bromodeoxyuridine/5-bromo-2’-deoxyuridine (BrdU) in the presence or absence of BPS (10 nM, 100 nM, 1 µM, 10 µM or 50 µM) and removal of the supernatant, cell proliferation was measured using an enzyme-linked immunosorbent assay (Cell Proliferation ELISA, BrdU [colorimetric], Roche Applied Science, Germany), according to the manufacturer’s recommendations. The absorbance was measured at 450 nm with a Thermo LabSystems plate reader (Thermo Fischer Scientific) and Ascent Software for Multiskan equipment. Cell proliferation was normalised to the control condition of each culture. The results are expressed as mean ± standard error of the mean (SEM) of five independent experiments with four replicates per condition.

### 4.6. Steroidogenesis Measurement

After 48-h treatment in the presence or absence of BPS (10 nM, 100 nM, 1 μM, 10 μM or 50 μM), supernatants and cells were separately stored at −20 °C until steroid and protein assays, respectively (see ‘Protein extraction and quantification’ below).

The progesterone concentration was determined in the culture media using a previously described ELISA [57]. The absorbance was measured spectrophotometrically at 405 nm with a Sunrise basic plate reader (TECAN Life Sciences, Switzerland) and Magellan software. For progesterone concentrations that ranged from 0.25 to 32 ng/mL, the intra-assay coefficient of variation (CV) averaged < 10%. Progesterone secreted in each well was normalised by the protein concentration of the same well. The results, expressed in ng progesterone per μg protein, were then normalised to the control condition of each experiment and presented as mean ± SEM of seven experiments with three replicates per condition.

The oestradiol concentration in the culture media was determined using the DIAsource E2-EASIA Kit (DIAsource, Louvain-la-Neuve, Belgium), in accordance with the manufacturer’s recommendations. Briefly, 50 μL of spent medium was used for the assay; the competition between unlabelled oestradiol (present in the culture media) and labelled oestradiol (provided by the kit) lasted 2 h at 4 °C. For oestradiol concentrations that ranged from 1.56 to 50 pg/mL, the interassay CVs averaged 15%. Data that represented seven independent cultures with each treatment conducted in duplicate are expressed as mean ± SEM as pg secreted oestradiol per μg protein. Oestradiol was normalised as described above for progesterone.

### 4.7. Protein Extraction and Quantification

After supernatant removal and addition of lysis buffer to the cells (150 mM NaCl, 1 mM Tris, 1 mM EDTA, 2 mM ethylene glycol tetraacetic acid [EGTA], 2 mM Na_3_VO_4_, 10 mM NaF, 12 mM NaH_2_PO_4_, 0.5% NP40 (*v/v*), 1% Triton X-100 [*v/v*]), proteins were extracted on ice. The recovered lysates were then centrifuged for 25 min at 16,000 *g*, and the supernatant protein concentration was measured using the BCA assay Protein Quantification colorimetric kit (Interchim, Montlucon, France), according to the manufacturer’s recommendations. Absorbance was measured at 550 nm with a Thermo LabSystems plate reader and Ascent software for Multiskan equipment.

### 4.8. Western Blot Analysis

Assays were conducted for steroidogenic enzymes after 48-h treatment in the presence or absence of BPS (10 μM or 50 μM): CYP11A1 and HSD3B1, enzymes related to progesterone secretion, and CYP19A1, related to oestradiol secretion. As mitogen-activated protein kinase 3/1 (MAPK3/1) has already been reported to mediate some BPA effects and because the MAPK3/1 pathway is involved in GC functions, its phosphorylation rate was investigated. MAPK 3/1 signalling pathway assays were conducted after 5, 10, 30 or 60 min in the presence or absence of 10 µM BPS. Protein was extracted from HGC as described above, and 8 µg protein lysates were subjected to western blotting, as previously described [58]. Blots were incubated with appropriate primary antibodies (Table 2) in tris-buffered saline tween 0.1% (TBST) with 5% non-fat dry milk powder (NFDMP) at 4°C overnight. After several washes in TBST, immunoreactivity was detected using anti-rabbit horseradish-peroxidase-conjugated secondary antibodies (diluted 1:10,000) and visualised using enhanced chemiluminescence (ECL; West Dura, Thermo Scientific, Courtaboeuf, France). A GeneGnome charge-coupled device camera (Syngene, Cambridge, United Kingdom) and Genesys 1.6.7 software (Syngene) was used to acquire the ECL signal. Signal intensity analysis was performed with GeneTools 4.01 software (Syngene). For steroidogenic enzyme assays, results are expressed in arbitrary units as the ratio of HSD3B1 (*n* = 5), CYP11A1 (*n* = 3) or CYP19A1 (*n* = 5) to vinculin and normalised to the control. For MAPK 3/1 signaling pathway assays (*n* = 6), the results are expressed as the ratio of phosphorylated to total protein signal intensity.

### 4.9. Gene Analysis

Six independent experiments with 3–4 HGC samples collected after 48-h treatment in the presence or absence of BPS (10 nM or 1 µM) were used for transcriptomic analysis using real-time quantitative polymerase chain reaction (qPCR). Briefly, total RNA was extracted from HCG using the TRI reagent and treated using XS RNA Nucleospin (Macherey Nagel, France), following the manufacturer’s instructions. Subsequently, the RNA concentration was determined using a NanoDrop ND-1000 spectrophotometer (Nyxor Biotech, Paris, France), and RNA integrity was checked using electrophoresis. DNAse treatment and reverse transcription (RT) was performed on 150 ng total RNA extracted from CC using the Maxima First Strand cDNA Synthesis Kit (ThermoFisher Scientific), according to the manufacturer’s recommendations.

qPCR reactions were performed as previously described [59]. The geometric mean of two housekeeping genes (ribosomal protein L19 [*RPL19*] and glyceraldehyde 3-phosphate dehydrogenase [*GAPDH*]) was used to normalise gene expression. The relative amounts of gene transcripts (R) were calculated according to the equation:

$$R\;=\;\frac{\left(E_{gene}^{-Ct\;gene}\right)}{\left(\mathrm{geometric}\;\mathrm{mean}\;\left(E_{GAPDH}^{-Ct\;GAPDH};\;E_{RPL19}^{-Ct\;RPL19}\right)\right)}$$

where E is the primer efficiency (Table 1) and Ct the cycle threshold.

### 4.10. Follicular Fluid Sample Collection and Analysis

Fifty-nine individual follicular fluid samples were retrieved during the IVF procedure and stored in glass tubes at −20 °C until assays. BPS glucuronide (BPSG) was quantified—without resorting to a hydrolysis step—using liquid chromatography-mass spectrometry with an Acquity U-HPLC device coupled to a Xevo-TQ triple quadrupole mass spectrometer (Waters, Saint-Quentin-en-Yvelines, France) operated with positive electrospray ionisation and MRM mode. Chromatographic separation was achieved on a Waters Phenyl-Hexyl U-HPLC column (2.1 × 100 mm; 1.6 µm) with an acidified H_2_O/AcN gradient elution (0.3 mL/min, 40 °C). Chromatographic data were monitored using Targetlynx® software (Waters Corporation). Briefly, the samples (250 µL) were purified with anionic exchange solid phase extraction (SPE) cartridges using BPS-G d8 (Toronto Research Chemicals) as internal standard. The resulting extract was derivatised with chloride dansyl. All the follicular liquid was quantified within 1 day with a calibration curve that ranged from 0.5 to 50 ng/mL. The accuracy and precision of the assay were evaluated with two series of three quality control (QC) samples at 0.75, 7.5 and 25 ng/mL. The mean accuracy and intra-day CV precision of the assay were 83% and 8%, respectively, and the limit of quantification (LOQ) was set at 0.5 ng/mL.

### 4.11. Statistical Analysis

Statistical analyses were performed with R version 3.5.2 software, using the R Commander package. For steroidogenesis, cell proliferation, viability (measured in supernatants) and signaling pathways, a nonparametric analysis of variance (ANOVA) by permutation (lmperm package) was performed due to a non-normal distribution (Shapiro test) and non-homogeneous variances (Levene test). Tukey’s post hoc test (nparcomp package) was used to determine differences between groups. Logistic regression analysis was used to analyse cell viability rates. The Wilcoxon test was used to analyse steroidogenesis protein expression. A *p* value ≤ 0.05 indicated significant difference, and 0.05 < *p* ≤ 0.10 indicated a tendency.

## 5. Conclusions

In the present study, we reported that BPS inhibited both progesterone and oestradiol secretion. BPS could partially affect progesterone secretion through a decrease in CYP11A1 expression. These effects may occur through distinct mechanisms and signalling pathways from those triggered by BPA. More importantly, BPS was detected in 18.6% of the follicular fluids recovered from women who were undergoing IVF procedure. This finding suggests that BPS may affect the success of their attempt.

## Figures and Tables

**Table 1:** Oligonucleotide primer sequences.

Abbrev.	Name	Forward (5′→ 3′)	Reverse (5′ → 3′)	Size (bp)	E (%)
*AR*	Androgen receptor	CTCTGGTGGTTCCCTCTCTG	AGCATCCAAGTGGCTTATGG	165	87.5
*CYP17A1*	Cytochrome P450 family 19 subfamily A member 1	TGAGTTTGCTGTGGACAAGG	TCCGAAGGGCAAATAGCTTA	163	100.4
*ESR1*	Oestrogen receptor 1	TCCAACTGCATTTCCTTTCC	TTGGAACATGGCAGCATTTA	201	82
*ESR2*	Oestrogen receptor 2	GATGCTTTGGTTTGGGTGAT	ATCGTTGCTTCAGGCAAAAG	175	98.9
*ESRRG*	Oestrogen-related receptor γ	AATGCTATCCTGCAGCTGGT	GCAGCGCTTCATGTAAGACA	159	94.7
*GAPDH*	Glyceraldehyde-3-phosphate dehydrogenase	GTCAGTGGTGGACCTGACCT	TGCTGTAGCCAAATTCGTTG	245	78.3
*GPER*	G protein-coupled oestrogen receptor	GCTGGTCTTCTTCGTCTGCT	GGTTTAGGCAGCTGTTGGAG	167	104.5
*PR*	Progesterone receptor	TCGAGCTCACAGCGTTTCTA	ACCATCCCTGCCAATATCT	183	76.5
*RPL19*	Ribosomal protein L19	CATGGAACACATCCACAAGC	TTGGTCTCTTCCTCCTTGGAT	171	92.5
*STAR*	Steroidogenic acute regulatory protein	CCTGAGCAGAAGGGTGTCAT	AGGACCTGGTTGATGATGCT	151	88.9

E: Efficiency of the primer pair.

**Table 2:** Characteristics of primary antibodies used for western blotting.

Name	Species Specificity	Source	Supplier (Distributor, Town, Country)	Catalogue Number	Primary Ab Dilution
MAPK3/1 (ERK1/2)	Rat	Rabbit polyclonal antibody	Cell Signaling Technology (Ozyme, Saint Quentin Yvelines, France)	9102	1/1000
Phospho-MAPK3/1 (ERK1/2; Thr202/Tyr204, D13.14.4E, XPTM)	Human	Rabbit monoclonal antibody	Cell Signaling Technology	4370	1/2000
CYP19A1	Human	Rabbit polyclonal antibody	Sigma Aldrich (Saint Louis, Missouri, USA)	SAB1410280X	1/500
CYP11A1	Human	Goat polyclonal antibody	Santa Cruz Biotechnology (Dallas, USA)	sc-18043	1/500
HSD3B1	Human	Rabbit polyclonal antibody	Abgent (San Diego, USA)	AP14585a	1/500
Vinculin	Human	Mouse monoclonal antibody	Sigma Aldrich (Saint Quentin Fallavier)	V9131	1/1000
